# The Impact of Two-Invoice System on Pharmaceutical Manufacturers’ Selling Expenses in China: A Difference-in-Differences Approach

**DOI:** 10.3390/ijerph19074400

**Published:** 2022-04-06

**Authors:** Yi Ran, Yuanyuan Hu, Shouming Chen, Fangjun Qiu, Ahmed Rabeeu

**Affiliations:** 1 School of Economics and Management, Tongji University, Shanghai 200092, China; ranyi@shaphar.com (Y.R.); qfjdyx@163.com (F.Q.); rabeeu@tongji.edu.cn (A.R.); 2 SPH KDL Health Chongqing Pharmaceutical Co., Ltd., Chongqing 400050, China

**Keywords:** two-invoice system, pharmaceutical industry, pharmaceutical manufacturers’ selling expenses, drug distribution, DID

## Abstract

A perennial question for the pharmaceutical industry has been excessive drug prices. To alleviate patients’ burden of expensive medical bills and increase the affordability of medicines, China adopted the Two-Invoice System (TIS) in drug procurement for public medical institutions in 2017. In this paper, we study the impact of the TIS on pharmaceutical manufacturers’ selling expenses. Using a Difference-in-Differences (DID) methodology and a sample of the A-share pharmaceutical manufacturing firms listed on the Shanghai Stock Exchange and Shenzhen Stock Exchange from the years 2014 to 2020, we find that the TIS leads to a significant increase in pharmaceutical manufacturers’ selling expenses but gradually weakens over time. In addition, we further explore whether the impact of the TIS on pharmaceutical manufacturers’ selling expenses is affected by the pharmaceutical manufacturers’ previous drug circulation mode. The results indicate that the TIS could significantly increase the pharmaceutical manufacturers’ selling expenses in the agency mode group. However, there is no evidence to support the TIS having the same effect in the direct sales office model group.

## 1. Introduction

A perennial question for the pharmaceutical industry has been the excessive drug prices [1,2,3,4,5,6,7,8,9]. Drug price has a direct impact on affordability and access to drugs, particularly in countries where out-of-pocket spending accounts for a large share of pharmaceutical spending, such as in China [10]. Recent years have seen an increase in criticism regarding the rising cost of certain drugs. One result of this public clamor is that the pricing structure of this industry has once again been scrutinized by government authorities and the media. Without a doubt, excessive drug prices have become a critical issue in accessing healthcare. Controlling high drug prices is an important objective for healthcare policymakers [11].

It is sometimes claimed that these exorbitant charges are unjustifiable. It is believed that pharmaceutical companies could supply less expensive drugs. Possible explanations include the complicated market structure or information asymmetry, as well as the division of duty between patients and purchasing decision makers who bear the expense [10,12,13]. For instance, some scholars argue that drug prices in the United States are significantly higher than in other countries as a result of the free market in America and the oligopolistic nature of the pharmaceutical industry [12]. Using case studies from Australia, China, India, Malaysia, New Zealand, and South Korea, some experts believe that the absence of more affordable medicine pricing is due to insufficient competition [10]. In China, a significant amount of the total costs of drugs is derived mainly from commercial promotion activities and profits generated by numerous levels of distribution [10,14]. As a result, many opponents argue that the way to rectify this inequity may be through government regulation.

In practice, authorities have attempted to implement a variety of reformatory pharmaceutical pricing policies in order to reduce patients’ burden of high medical costs and raise the affordability of medicines [10]. China is no exception [14,15,16,17,18,19,20]. On 9 January 2017, the Medical Reform Office of the State Council, in collaboration the National Health and Family Planning Commission of the People’s Republic of China, the China Food and Drug Administration, the National Development and Reform Commission, the Ministry of Industry and Information Technology of the People’s Republic China, the Ministry of Commerce, the State Administration of Taxation, and the State Administration of Traditional Chinese Medicine, issued the notice on the implementation opinions of the Two-Invoice System (TIS) in drug procurement for public medical institutions. The purpose of this notice is to reduce the links of drug circulation, to make the middle price transparent, to further promote the reduction in falsely high drug prices, and to alleviate the burden of drug use [21]. The TIS refers to the mechanism where only up to two invoices are issued along the chain of pharmaceutical product procurement, with one issued by the pharmaceutical manufacturer and the only other issued by the distributor to the medical service providers, which is an important reform in drugs circulation.

The influence of TIS has garnered increasing academic attention [17,18,19,20]. Not only is the TIS reform designed to lower drug costs, but also to attempt to cut drug prices by minimizing the links in drug circulation, thereby promoting the development of the sector of drug circulation. However, it has been discovered that implementing the TIS policy has a detrimental effect on the performance of pharmaceutical firms. Pharmaceutical marketing is one of the important ways for pharmaceutical firms to create value. Clearly, the TIS policy has shortened the marketing channels of pharmaceutical manufacturers, which may result in lower drug prices. What impact will it have on the marketing activities of pharmaceutical manufacturers? If the TIS policy significantly increases the selling expenses of pharmaceutical manufacturers, the effect may be the inverse of what was anticipated. This is because a portion of the price increases are passed on to consumers. The majority of research conducted in the aftermath of the implementation of the TIS policy has concentrated on the influence of the policy on drug pricing, patient medication, and the pharmaceutical industry. Additionally, a few empirical studies have systematically investigated the relationship between the TIS reform and the selling expenses of pharmaceutical companies. Therefore, focusing on this subject has practical implications at the level of institutional design, providing policy guidance for the institutional reform of drug marketing channels but also theoretical implications at the level of academic research, supplementing empirical evidence on the relationship between institutions and corporate marketing activities.

Our study aims to investigate the impact of the TIS reform on pharmaceutical manufacturers’ selling expenses, which are defined as the costs associated with distributing, marketing, and selling a product or service. This study employs the Difference-in-Difference (DID) approach to examine the effect of the TIS on pharmaceutical manufacturers’ selling expenses. It is based on assessments of a sample of A-share pharmaceutical companies listed on the Shanghai and Shenzhen stock markets. Our study makes three contributions. First, this paper expands the literature on pharmaceutical policy in the emerging economy by conducting the first empirical examination of the effect of China’s TIS on pharmaceutical manufacturers’ selling expenses. Second, we employed DID methodology to alleviate some of the endogenous problems of general regression analysis and increase the credibility of the research conclusion [22]. Finally, this study also contributes to the marketing studies by revealing the impact of the TIS on the selling expenses of pharmaceutical manufacturers depending on the previous drug circulation mode.

This paper proceeds as follows. In Section 2, we review the institutional background of the TIS and develop the hypothesis. Section 3 describes our data collection procedure and variable construction and outlines the empirical method used to investigate the hypothesis. Section 4 presents the results and a discussion of the empirical analysis. Section 5 concludes by offering discussion, interesting implications, and the limitations of the findings.

## 2. Theory and Hypothesis

### 2.1. Instituational Background the Two-Invoice System

The most prominent feature of the Chinese pharmaceutical industry’s marketing channels is multiple layers of distribution [10]. The pharmaceutical supply chain includes manufacturing, distribution, wholesalers, retailers, hospitals, and patients [14].

Previously, under the Multi-Invoice System, pharmaceutical manufacturers could sell their products directly to hospitals in addition to wholesalers and retailers. Meanwhile, larger distributors can sell to smaller distributors. In other words, the pharmaceutical distribution sector is highly fragmented due to the presence of several distribution layer [10,14,15]. There were over 13,100 distributors in 2017, and the top four Chinese distributors controlled 37.6% of the market. As a result of this fragmentation, various purchase quantities may result in different wholesale prices, resulting in a significant imbalance within the pharmaceutical distribution industry. Additionally, drugs were distributed through multiple layers of distribution before reaching the patient, resulting in inefficiencies and higher drug prices [10]. On the other hand, pharmaceutical manufacturers have a strong motivation to actively promote their drugs through commercial promotion efforts and profit sharing with various levels of distribution. For instance, advertising for competing over-the-counter drugs or employing a large number of salespersons or medical representatives to promote prescriptions [10].

Therefore, prior to the implementation of TIS, the pharmaceutical industry’s marketing channels were extremely complex, with a mix of distributions, wholesalers, and retailers, significantly increasing the difficulty of supervision in terms of relevant departments’ direct supervision capability and the degree of information opacity [11,16,18,19]. Simultaneously, the multi-invoice system makes it more difficult to improve the industry’s concentration, and the fragmented market environment exacerbates the difficulty of supervision [11]. With the help of the multiple layers of distribution, manufacturing firms can entrust the selling expenses to the distributions (e.g., pay the commission fee to the distributions) by transferring profits at a low price, thereby obtaining a sufficient profit while avoiding supervision. Thus, all these contribute to a substantial proportion of the total costs of drugs.

The Chinese government has reformed the pharmaceutical distribution network in order to decrease drug circulation links, make the middle price increase transparent, promote the reduction in high medicine prices, and alleviate the burden of drug use. On 9 January 2017, the Medical Reform Office of the State Council, in collaboration with the other seven agencies, issued a notice regarding the implementation opinions of the TIS in drug procurement for public medical institutions. This notice clearly defines TIS, which means that two invoices are issued along the chain of pharmaceutical product procurement, with one issued by the pharmaceutical manufacturer and the other issued by the distributor to the medical service providers. In addition, the notice requires public medical institutions to gradually implement the TIS in drug procurement, while encouraging other medical institutions to do so [21]. By the end of 2017, all 31 provinces, municipalities, and autonomous regions on the Chinese mainland have formally released a notification on the TIS ‘s implementation opinions. According to collected data, a total of 30 provinces began implementing the TIS formally by the end of 2017, and the whole Chinese mainland began doing so in 2018, with the exception of 1 province.

Accordingly, the implementation of TIS forces the pharmaceutical industry to reduce redundant distributions in marketing channels, directly cutting off the multi-invoice marketing channels, and has a huge lash on the marketing channels of the pharmaceutical distribution industry.

### 2.2. The Two-Invoice System and Pharmaceutical Manufacturers’ Selling Expenses

Pharmaceutical marketing is widely recognized as a critical channel for pharmaceutical makers to create value. Pharmaceutical firms can create value by increasing product popularity and market share through marketing. Distributors benefit from a comparative advantage in marketing activities when using the Multi-Invoice System. As a result, pharmaceutical producers can outsource sales to distributors by sacrificing profits at a low price, lowering the gross profit margin of drugs [10,14]. Correspondingly, the book-selling expenses of pharmaceutical manufacturers are likewise relatively low.

However, the TIS highlights the hidden selling expenditures and increases the transaction costs of the pharmaceutical manufacturers. Firstly, as mentioned above, the purpose of the implementation of the TIS is to make the intermediate price increase transparent, to promote the reduction in falsely high prices of drugs, and to minimize the burden of drug use by reducing the links of drug circulation. The implementation of the TIS directly influenced the drug marketing model of pharmaceutical manufacturers, so that pharmaceutical manufacturers had to integrate the multi-level marketing channels under the original multi-invoice system into one level, and even afford some marketing activities by themselves. According to some studies, only about 12.5% of pharmaceutical manufacturers in China had established marketing teams prior to the implementation of TIS. Due to the limited human and financial resources, the majority of pharmaceutical manufacturers distribute their products via marketing networks and agents. As a result of the TIS implementation and channel compression, pharmaceutical manufacturers must expand downstream of the value chain. Therefore, selling expenses will increase rapidly.

In conclusion, we propose that the TIS highlights the hidden selling expenses and increases the transaction costs of the pharmaceutical manufacturers. These conclusions lead us to the following hypothesis:

**Hypothesis 1.**  *The implementation of the TIS will increase the selling expense of pharmaceutical manufacturers*.

## 3. Research Design

### 3.1. Sample Selection and Data

The initial sample of this study included all the A-share listed pharmaceutical manufacturing firms on the Shanghai Stock Exchange and Shenzhen Stock Exchange. From 2014 to 2020, our sample consisted of an unbalanced panel. The selection procedures are as follows: (1) This paper excludes the observations made in 2017 to ensure the accuracy of policy effect identification. Since the TIS began to be implemented in all provinces in early 2017 and was officially implemented nationwide in 2018, many regions were in the process of transiting from the Multi-Invoice System to the Two-Invoice System in 2017. (2) This paper excludes sample companies listed after 2014, since the calculation of treated variables requires the data from both the control and the treatment groups at the same time. (3) This paper also excludes the sample companies with incomplete data. Finally, a total of 1198 “firm-year” observations were obtained over a 6-year period. Accounting statement and selling expenses data of listed companies were sourced from the CSMAR database and Wind databases [23]. The sequence data of implementing the TIS in each region were manually gathered from the official websites of the governments of each region and other relevant websites.

### 3.2. Regression Method

This study aims to investigate the effect of the TIS on pharmaceutical manufacturers’ selling expenses. The reform of the TIS can be viewed as a quasi-natural experiment. By comparing the treatment and the control groups, we can determine the net effect of the TIS. Nevertheless, the difference between the treatment and the control groups could be explained by other unobservable factors that do not change over time. Thus, direct comparison is bound to cause endogenous issues [24,25]. In this case, the DID technique was used to estimate the real effect of the TIS on pharmaceutical manufacturers’ selling expenses. Finally, we established the following regression model as a benchmark:

(1) $${\mathrm{lnSE}}_{i,t}=\beta_{0}+\beta_{i}+\beta_{t}+\beta_{1}DID+\beta_{k}\sum_{k=2}^{10}Controls_{i,t-1}+\varepsilon_{i,t-1}$$

In Equation (1), *i* refers to firms, *t* refers to years, ln${\mathrm{SE}}_{i,t}$ refers to the natural log of selling expenses of firm *i* at year *t*, and $\beta_{i}$ and $\beta_{t}$ are the year and firm fixed effects, respectively. DID  is the “treatment dummy”—i.e., a dummy variable that equals 1 if the pharmaceutical firm belongs to pharmaceutical manufacturers which are directly affected by the TIS at year t. $\sum_{k=2}^{10}Controls_{i,t-1}$ is the vector of control variables of the firm *i* at year *t* − 1, which includes ROA, Tobin’sq, leverage, slack resource, size, age, ownership, the shareholding ratio of the largest shareholder, and separation ratio. All control variables are lagged by one year. $\beta_{0}$ is an intercept term,$\varepsilon_{i,t-1}$ is an error term. Ordinary least squares (OLS) are used to estimate the regression. To account for the serial correlation of the error term, we cluster standard errors at the firm level. The coefficient of interest measures the effect of the TIS on pharmaceutical manufacturers’ selling expenses. The hypothesis predicts that it should be positive and significant.

### 3.3. Variables

#### 3.3.1. Dependent Variable

Selling expenses. The costs of distributing, marketing, and selling a product or service are referred to as selling expenses. Selling expenses primarily include advertising, promotional and selling expenses, salaries and benefits for sales and marketing, and the other expenses raised by marketing activities for selling produce or service including depreciation, repairs, supplies consumed, amortization of low-cost and short-lived articles, office expenses, traveling, commission, consignment handling fees, transportation, loading and unloading, packaging, insurance, rental, sales service fees, and miscellaneous. To enhance normality and correct positive skewness, log transformation is employed for selling expenses. Following the common practice in the literature, we used the logs of the selling expenses as the dependent variable. During the robustness test, we also used the selling expenses intensity(selling expenses/sales) as an alternative measure [26].

#### 3.3.2. Independent Variable

Treatment dummy. This dummy variable is to define whether a firm is a treatment firm in a certain year. China began to implement the TIS in 2017. By 2018, the TIS had been fully implemented in the pharmaceutical industry. As a result, 2017 can be considered a transitional year for the nationwide adoption of TIS. This paper excludes the observation in 2017 in order to accurately identify the treatment with the effect of the TIS. We constructed a time dummy variable (Post) to indicate whether the observation occurred prior to or following 2017. The Post is equal to 1 if the observation occurs between 2018 and 2020. Otherwise, the Post is equal to 0.

According to the general DID model, the treatment should accurately distinguish the treatment group affected by the policy from the control group that is not influenced by the policy. The TIS studied in this paper applies to the pharmaceutical industry. However, not all firms in the pharmaceutical industry are equally affected. As previously stated, because the TIS is primarily directed at drug circulation, pharmaceutical manufacturing firms that are not involved in this field are less or not affected, allowing this study to distinguish between the treatment and control groups. We divided the pharmaceutical industry into two groups (treatment and control group): those directly impacted by the policy and those whose principal source of revenue is medical machinery production, real estate development, and other medical devices. The major business revenue criteria are based on the composition of the primary business revenue revealed in the annual report. For treatment group samples, the dummy variable, Treat, equals 1, and for the control group, the dummy variable, Treat, equals 0. The final independent variable, DID, is determined through an interaction between the treatment group dummy variable (Treat) and the time dummy variable (Post).

#### 3.3.3. Controls Variables

We controlled for a vector of firm-level characteristics that may affect selling expenses in our baseline regressions (see Section 3.2). All control variables are obtained from the CSMAR database and the Wind database. Specifically, we controlled for ROA, Tobin’s Q, leverage, slack resources, size, age, ownership, the shareholding ratio of the largest shareholder, and the separation ratio. In auxiliary analysis, we further examine whether the effect of TIS on pharmaceutical manufacturers’ selling expenses varies according to firms’ drug circulation mode [14]. To distinguish the drug circulation mode between the direct marketing model and the agency mode, we used the selling expenses rate to measure the distribution mode of pharmaceutical manufacturers. Specifically, this paper divides the sample firms into a direct marketing mode group and an agency mode group depending on the size of their selling expenses rate prior to the implementation of the TIS. See Appendix A for further details.

## 4. Results

### 4.1. Descriptive Statistics

Table 1 and Table 2 present the descriptive statistics and the correlations of the variables of this study. As shown in the table, the mean value of selling expenses for the sample is 19.42, and the standard deviation of selling expenses is 1.561. The coefficient of variation is greater than 5, indicating the selling expenses raised by the TIS reform differ between pharmaceutical firms. Additionally, the max value of the selling expenses intensity of the sample is 74.4%, while the min value of the selling expenses intensity of the sample is 0.3%, suggesting that selling expenses vary across pharmaceutical companies.

Table 2 lists the correlations of all variables. It seems that some variables were highly correlated with each other, suggesting the possibility of a multi-collinearity problem. We used variance inflation factors (VIFs) to check for multi-collinearity and found that the largest variance inflation factor (VIF) is 2.33 and the average value is 1.64. All of the VIFs are far below the rule-of-thumb cutoff of 10. Therefore, no evidence of multi-collinearity exists, and the variables are now suitable for further study.

### 4.2. The Results of DID

#### 4.2.1. The Impact of the Two-Invoice System on Selling Expenses

Table 3 presents the stepwise regression results using DID methodology. From model 1 to model 2 and model 3 to model 4, we incrementally added DID and control variables. Our study is to investigate the influence of the TIS on selling expenses. As Table 4 shows, the results of model 2 and model 4 preliminarily show that the coefficient of DID is significantly positive at the confidence levels of 1% (β = 0.5018, *p* < 0.01) and 5% (β = 0.3836, *p* < 0.05), respectively. In other words, the results show that following the TIS policy, the natural log of selling expenses of pharmaceutical manufacturers has increased by 1.96% (0.3836/19.42), which indicating that the TIS policy raised the selling expenses of pharmaceutical manufacturers. Table 3 shows that regardless of whether the time fixed effect is controlled or not, the interaction term is significantly positive to the selling expenses, indicating the robustness of the results. Therefore, the hypothesis is supported.

#### 4.2.2. Dynamics of the Two-Invoice System Effect

In Table 4, we assessed the dynamics of the treatment effect. To accomplish this, we replaced the treatment dummy variable with a set of five dummy variables representing the three years preceding the treatment (pre_3 and pre_2); the year of the treatment (current); and the first, second, and third years following the treatment (post_1, post_2, and post_3, respectively). As shown, the coefficients of all pretreatment dummies are small and insignificant, whereas the coefficients of the current and posttreatment dummies are large and significant, indicating that there is no preexisting trend in the data and our sample satisfies the parallel trend test. Interestingly, we find no effect in the third year following the treatment, and the coefficient of post_3 is decreased and insignificant. As shown by the positive and significant coefficients of current, post_1 and post_2, the TIS had an influence on selling expenses before and after the treatment year, although the effect eventually decreased. This shows that the effect of the TIS on increasing selling expenses of pharmaceutical manufacturers is temporary, lasting approximately 12 to 24 months. In other words, the TIS has no long-term impact on selling expenses.

**Figure 1:**
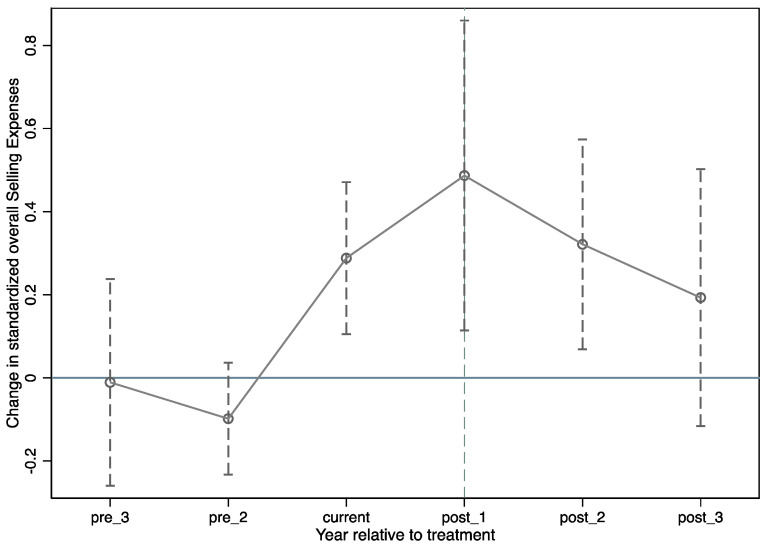
The Dynamic Impact of Two-Invoice System on Selling expenses.

In Figure 1, we plot the coefficients of the above dummy variables (pre_3, pre_2, current, post_1, post_2, and post_3) in Table 5 and the 95% confidence intervals. As shown in Figure 1, selling expenses are trending upward in both the control and treatment groups. This demonstrates the importance of using a control group—not accounting for changes in selling expenses in the control group would overstate the effect of the TIS on selling expenses, because it would capture some of the time trends. Overall, the patterns in Figure 1 mirror the patterns in the models (1) and (2) of Table 5. In particular, since there is no preexisting trend, the effect gradually diminishes over time; specifically, the increase in selling expenses gradually diminished with time.

### 4.3. Auxiliary Results

The TIS reform mainly occurs in the field of drug circulation. In the process of circulation, it is necessary to issue invoices twice, one is for drug manufacturers to issue invoices to distribution firms, and the other is for distribution firms to issue invoices to medical institutions [17,18,19,20]. It is indicated that the effect of the TIS on pharmaceutical manufacturers’ selling expenses depends on the firms’ previous drug circulation mode. Therefore, it is necessary to explore whether the TIS has a differential effect on pharmaceutical manufacturers’ selling expenses with varying prior drug circulation mode.

There are several drug circulation modes, including direct to pharmacy distribution (DTP) via a single agency, reduced wholesaler models (RWM), and treating wholesalers purely as logistic providers. In China, there were two traditional drug circulation modes [5]. The first is comparable to the DTP model, which is characterized as the direct sale office model (DSOM). Pharmaceutical manufacturers have formed not only their own sales teams and publicity teams but also a promotion team in the DSOM model. The other is comparable to the RW and is referred to as the agency model (AM). Pharmacy sales originate from (full-line) wholesalers in this drug circulation mode. Meanwhile, wholesalers are responsible for market development, academic promotion, and product publicity [14]. Whichever circulation mode is used to bring drugs to market is decided by the manufacturer’s strength (operation level, production level, sales level, monopoly level, etc.). We use the selling expenses rate to determine the distribution mode of pharmaceutical firms and distinguish the drug circulation mode between the DSOM and AM. Specifically, we divide the sample firms into a DSOM group and an AM group depending on the size of selling expenses rate prior to the implementation of the TIS. If the selling expenses rate is greater than or equal to the industry average and median, the company is classified as the DSOM group; otherwise, it is classified as the AM group.

Finally, we adopt a DID model to explore the different effects of the TIS on pharmaceutical manufacturers’ selling expenses in two groups. In Table 5, the results are shown that the coefficients of the DID in the DSOM group are insignificantly negative (β = −0.1342, *p* > 0.1) in model 1 and significantly negative (β = −0.2734, *p* < 0.01) in model 2. However, the corresponding coefficients of DID are significantly positive in the AM group (β = 0.2977, *p* < 0.1, model 3; β = 0.3506, *p* < 0.1, model 4). These results indicate that the TIS could significantly increase the pharmaceutical manufacturers’ selling expenses in the AM group. However, there is no indication that TIS has the same effect in the DSOM group. In other words, the impact of the TIS on pharmaceutical manufacturers’ selling expenses is indeed affected by the pharmaceutical manufacturers’ previous drug circulation mode.

The results could be explained by the fact that, prior to the introduction of the TIS, only about 600 of China’s over 4800 pharmaceutical companies had established marketing teams. Due to limited human and financial resources, the majority of pharmaceutical manufacturers distribute their products through agency mode due. Affected by the AM, the price given by pharmaceutical manufacturers to agents is the cost price, and the agents are responsible for the entire link. On the one hand, this mode not only opens the market rapidly but also quickly recovers funds, thereby improving market coverage. On the other hand, public relations, promotion, and marketing are all expensive. At the same time, there are too many distributors and too many price increases, which raises the cost of pharmaceuticals and jeopardizes their quality. As mentioned before, the purpose of the TIS is to reduce the high drug prices by compressing the circulation link. Therefore, the TIS has a small effect on firms with high ex-factory prices but a substantial effect on firms with low ex-factory prices. In other words, the TIS has little impact on pharmaceutical manufacturers’ selling expenses with the DSOM but has a significant effect on pharmaceutical manufacturers’ selling expenses with the AM.

### 4.4. Robutenss Check

#### 4.4.1. Placebo Test

To address the issue that the documented effect could be driven by spurious correlations in our data, we conducted placebo tests by advancing the time window of the TIS reform by 3 years [25]. The results are shown in Table 6. The results show that the coefficients of DID are not statistically significant regardless of whether the year fixed effect is controlled or not, implying that the results of the main effect are no longer present in the absence of TIS implementation and passing the placebo test.

#### 4.4.2. Multi-Period DID

When collecting data, we discovered that, while the TIS was officially implemented at the central level on 26 December 2016, there were variances in the actual implementation time points of the TIS in various regions. These observations are more pronounced between 2014 and 2018. For instance, Fujian has been implementing it since 25 June 2014, Chongqing since 31 December 2016, Shanxi since 1 January 2017, and the Tibet Autonomous Region has been implementing it throughout the province since 1 January 2018. This enables the construction of a new DID model for testing the robustness the TIS in the order in which it is actually implemented in the province [27]. Specifically, if the sample firm has implemented the TIS in the current year, it is assigned to the treatment group. The value of the Post variable is 1, and the value of post in subsequent years is also 1, whereas the value of post in previous years is 0. The value of the Treat variable is the same as before, and the definitions of the other variables are consistent with the main effect. The regression results are shown in Table 7. The coefficients of DID are 0.3569, 0.4140, and 0.4140, and are statistically significant at the levels of 5%, 10%, and 10%, respectively. It is consistent with the results of the main test.

#### 4.4.3. Alternative Dependent Variable

In our baseline specification, we used the logs of the selling expenses as the dependent variable. However, a drawback is that selling expenses may be affected by firm size. To address this issue, we re-estimated our baseline specification using the selling expenses intensity (marketing expense/sales) as an alternative dependent variable [26]. As is shown in Table 8, the coefficients of DID are 0.0790 and 0.0768, respectively, and are statistically significant at the levels of 1%. This indicates that our results are robust to the main test.

#### 4.4.4. PSM-DID

Another potential problem is that unobservable characteristics between the treatment group and control group may differ. To mitigate this issue, we employed a matching approach—i.e., we established a sample of matched control firms that are similar to the treated firms ex ante. We followed the matching algorithm described in previous literature [27,28]. First, for each treated firm, we considered pharmaceuticals that belong to production firms. We selected a candidate from the pool of candidates using one to four nearest-neighbor-matching, caliper-matching, and kernel-matching methods [29] based on the following firm-level characteristics: ROA, Tobin’s Q, leverage, slack resource, size, age, ownership, the shareholding ratio of the largest shareholder, and the separation ratio. Additionally, considering that this study contains data points from multiple years and following the previous studies [27,30], we employed a year-by-year matching method based on the three above methods. For each treated firm and each matched control firm, we compute the log(1 + selling expenses) in the three years following the treatment and the corresponding value in the three years preceding the treatment. The value of the DID variable is the same as before, and the definitions of the other variables are consistent with the main effect. The difference is then regressed on the treatment dummy. The results are provided in Table 9, the coefficients of DID are 0.2987,0.3236, 0.2773,0.2969, 0.4587, and 0.3756, respectively, which are significant at the levels of 10%, 10%, 10%, 10%, 1%, and 5%, respectively. It means that our results are robust to the main test.

#### 4.4.5. Confounding Effects

Another potential concern is that the increase in selling expenses in the post-treatment period may be due to confounding factors [28]. It is possible that our findings are due to the fact that treated and control firms differed throughout the COVID-19 epidemic. To address this issue, we re-estimated our baseline specification by excluding the samples corresponding to the year 2020. As shown in Table 10, our results remain unchanged and the coefficients of DID are 0.3689 and 0.4481, respectively, which are significant at the levels of 1% and 5%.

## 5. Conclusions

This study investigates whether the major policy of the pharmaceutical industry, the TIS reform, increases the selling expenses of pharmaceutical manufacturers. We used the DID approach to empirically examine the effect of the TIS reform on pharmaceutical firms’ selling expenses both before and after the policy’s implementation. The results indicate that implementing the TIS increases pharmaceutical manufacturers’ selling expenses significantly, although the rise steadily diminishes over time. Additionally, we further explore whether the impact of the TIS on pharmaceutical manufacturers’ selling expenses is affected by the pharmaceutical manufacturers’ previous drug circulation mode. The findings suggest that the TIS has the potential to significantly increase pharmaceutical manufacturers’ selling expenses in the agency mode group. However, there is no evidence to support the TIS having the same effect in the direct sales office model group.

These results are counterintuitive, as most views believe that China’s implementation of the TIS can reduce the falsely high drug prices by reducing the overall distribution cost of the whole pharmaceutical industry [17,18,19,20]. Because the TIS shortens the drug marketing channels, improves the transparency of drug marketing channels, and then improves the government’s ability to supervise drug marketing channels. However, the implementation of the TIS makes the pharmaceutical manufacturers assume marketing and sales functions, which highlights the hidden selling expenses of the traditional pharmaceutical distribution system. Thus, in the short term, it increases the selling expense of pharmaceutical manufacturers. However, this does not mean that the TIS policy is ineffective. Our results show that while the TIS policy initially raises pharmaceutical manufacturers’ selling expenses, this effect does not persist in the long run. It is indicated that TIS will gradually cut drug prices. Although the selling expenses of pharmaceutical manufacturers will increase in the short term, in the long run, the TIS policy standardizes the circulation of pharmaceutical products, purifies the pharmaceutical market environment, and promotes the long-term development of pharmaceutical manufacturers.

Additionally, it should be noted that initial stage of policy implementation may result in an increase in pharmaceutical makers’ selling expenses, posing tax-related risks. For example, a large number of CSOs (contract sales organizations), which are professional institutions that provide customers with market research, product design, and promotion services, are pouring out, and the authenticity and rationality of the cooperation between the pharmaceutical manufacturers and these CSO companies has become critical to the tax-related risks of pharmaceutical manufacturers. In this vein, the government should take other auxiliary measures to cooperate with the implementation of the TIS policy, such as improving the drug approval system and promoting the implementation of “consistency evaluation” of generic drugs, so that the selling expenses can be reduced, and doctors will no longer be required to listen to the opinions of agents to ensure the quality and efficacy of drugs.

Our study has the following implications. First, this paper expands the literature on pharmaceutical policy in the emerging economy by conducting the first empirical examination of the effect of China’s TIS on pharmaceutical manufacturers’ selling expenses. Second, our study advances evidence-informed and evidence-based policymaking [31,32]. A policy can be strongly evidence-informed if its advocates act effectively and empirical studies help explain when, how, and why such a policy works in a given context. In this study, we demonstrated empirically when, how, and why the TIS affects pharmaceutical manufacturers’ selling expenses. On the other hand, by evaluating the success of the TIS policy in the pharmaceutical industry, this study assists the government in making pertinent counselling policy decisions in the future. Finally, this study also contributes to the marketing studies by revealing the impact of the TIS on the selling expenses of pharmaceutical manufacturers depending on preceding drug circulation mode.

This study also has some limitations. Firstly, this study focused exclusively on the direct impact of the TIS policy on pharmaceutical manufacturers’ selling expenses. In fact, there is an indirect impact of the TIS policy on the innovation investment of pharmaceutical manufacturers. Future studies should focus on the impact of the TIS policy on the innovation of pharmaceutical manufacturers since innovation is critical for pharmaceutical companies [33]. Secondly, when considering the moderator variables, this study only focuses on the impact of the drug circulation mode. Actually, the mode of corporate value creation (marketing-orientation versus innovation-orientation) will also have an effect the relationship between the TIS policy and pharmaceutical manufacturers’ selling expenses. Additional moderators can be added in future studies to better investigate the moderating effect. Finally, the TIS policy has a considerable impact on the entire sector of drug circulation. In this study, we only focus on the pharmaceutical manufacturers, but the influence of TIS policy on agents and intermediates, such as CSO firms, can be considered in future studies.

## Figures and Tables

**Table 1:** Descriptive Statistic.

Variables	N	Mean	Sd	Min	Max
1. lnSE	1198	19.42	1.561	13.82	22.81
2. SE intensity	1198	0.248	0.174	0.003	0.744
3. Post	1198	0.601	0.49	0	1
4. Treat	1198	0.915	0.279	0	1
5. ROA	1198	0.058	0.079	−0.389	0.282
6. Tobin’s Q	1198	2.751	2.132	0.837	16.86
7. Lev	1198	0.309	0.178	0.035	0.886
8. Slack	1198	−0.006	0.115	−0.43	0.339
9. SIZE	1198	21.8	1.056	19.15	24.73
10. AGE	1198	10.47	7.509	0.063	27.16
11. Ownership	1198	0.178	0.383	0	1
12. BIGR	1198	33.27	12.79	7.77	69.16
13. Seperation	1198	4.88	7.149	0	29.83

**Table 2:** Correlations.

Variables	1	2	3	4	5	6	7	8	9	10	11	12
1. lnSE	1											
2. SE intensity	0.520 ***	1										
3. Post	0.174 ***	0.208 ***	1									
4. Treat	0.096 ***	0.149 ***	0.008	1								
5. ROA	0.127 ***	−0.051 *	−0.111 ***	−0.070 **	1							
6. Tobin’s Q	−0.217 ***	−0.064 **	−0.219 ***	−0.087 ***	0.249 ***	1						
7. Lev	0.088 ***	−0.179 ***	−0.01	0.085 ***	−0.387 ***	−0.089 ***	1					
8. Slack	0.205 ***	0.024	0.148 ***	0.029	0.195 ***	0.068 **	−0.002	1				
9. SIZE	0.667 ***	−0.062 **	0.134 ***	0.111 ***	−0.084 ***	−0.276 ***	0.291 ***	0.185 ***	1			
10. AGE	0.356 ***	−0.021	0.039	0.124 ***	−0.141 ***	−0.026	0.334 ***	0.230 ***	0.497 ***	1		
11. Ownership	0.177 ***	−0.131 ***	−0.058**	0.134 ***	−0.012	−0.083 ***	0.140 ***	0.121 ***	0.254 ***	0.373 ***	1	
12. BIGR	0.115 ***	0.011	−0.045	0.059 **	0.181 ***	−0.002	−0.118 ***	0.053 *	0.089 ***	−0.142 ***	0.182 ***	1
13. Seperation	0.189 ***	0.098 ***	−0.033	0.154 ***	0.038	0.006	0.012	0.083 ***	0.184 ***	0.278 ***	0.084 ***	0.157 ***

Notes: significance levels: * *p* < 0.05; ** *p* < 0.01; *** *p* < 0.001.

**Table 3:** The Impact of Two-Invoice System on Selling expenses.

	(1)	(2)	(3)	(4)
Variables	lnSE	lnSE	lnSE	lnSE
DID	0.8240 ***	0.5018 ***	0.3071 *	0.3836 **
	(0.064)	(0.076)	(0.172)	(0.159)
ROA		0.8966 **		0.9773 ***
		(0.393)		(0.373)
Tobin’s Q		−0.0200 *		−0.0014
		(0.012)		(0.012)
Lev		0.9013 ***		1.0538 ***
		(0.256)		(0.254)
Slack		0.0506		0.0668
		(0.169)		(0.168)
SIZE		0.3723 ***		0.3134 ***
		(0.094)		(0.096)
AGE		0.0250		−0.7744 *
		(0.020)		(0.447)
Ownership		0.2512		0.2951 *
		(0.171)		(0.172)
BIGR		0.0029		0.0024
		(0.007)		(0.007)
Separation		0.0049		0.0032
		(0.007)		(0.007)
_cons	18.9686 ***	10.3288 ***	18.8027 ***	17.0287 ***
	(0.035)	(1.921)	(0.053)	(3.804)
Firm FE	Yes	Yes	Yes	Yes
Year FE	No	No	Yes	Yes
N	1198	1198	1198	1198
R2	0.398	0.465	0.441	0.489
adj. R2	0.398	0.460	0.438	0.482

Notes: This table presents regression results of the interaction term (DID) on the natural log of selling expenses in the same regression sample in Columns (1) to (4), respectively. From Column (1) to Column (2) and Column (3) to Column (4), we add DID and control variables, in turn. Standard errors are clustered by firm level. ***, **, and * denote significance at 1%, 5%, and 10% level, respectively.

**Table 4:** The Dynamic Impact of Two-Invoice System on Selling expenses.

	(1)	(2)
Variables	lnSE	lnSE
pre_3	−0.0896	−0.0106
	(0.083)	(0.126)
pre_2	−0.1218 **	−0.0981
	(0.048)	(0.068)
current	0.2246 ***	0.2883 ***
	(0.050)	(0.093)
post_1	0.4042 ***	0.4871 **
	(0.083)	(0.190)
post_2	0.3883 ***	0.3215 **
	(0.109)	(0.128)
post_3	0.1587	0.1934
	(0.138)	(0.157)
ROA	0.8620 **	0.9340 **
	(0.367)	(0.367)
Tobin’s Q	−0.0055	−0.0017
	(0.013)	(0.012)
Lev	0.9211 ***	0.9594 ***
	(0.233)	(0.231)
Slack	−0.0347	−0.0347
	(0.144)	(0.142)
SIZE	0.2873 ***	0.2917 ***
	(0.084)	(0.084)
AGE	0.0731 **	−0.6034 *
	(0.033)	(0.333)
Ownership	0.2655	0.2783 *
	(0.166)	(0.168)
BIGR	0.0036	0.0035
	(0.007)	(0.007)
Separation	0.0036	0.0024
	(0.006)	(0.006)
_cons	11.7473 ***	16.2496 ***
	(1.738)	(3.102)
Firm FE	Yes	Yes
Year FE	No	Yes
N	1405	1405
R2	0.474	0.479
adj. R2	0.468	0.471

Notes: This table presents the dynamics of the treatment effect in the same regression sample in Columns (1) to (2), respectively. From Column (1) to Column (2), we add year fixed effect, in turn. Standard errors are clustered by firm level. ***, **, and * denote significance at 1%, 5%, and 10% level, respectively.

**Table 5:** Auxiliary Analysis.

Variables	DSOM Group		AM Group	
	(1)	(2)	(3)	(4)
	lnSE	lnSE	lnSE	lnSE
DID	−0.1342	−0.2734 ***	0.2977 *	0.3506 *
	(0.110)	(0.096)	(0.180)	(0.193)
ROA	1.5394 ***	1.5798 ***	0.6746	0.5138
	(0.561)	(0.547)	(0.443)	(0.465)
Tobin’s Q	0.0026	0.0011	0.0053	0.0080
	(0.008)	(0.008)	(0.016)	(0.017)
Lev	0.4414 *	0.4237 *	0.8335 ***	0.7421 **
	(0.264)	(0.248)	(0.313)	(0.323)
Slack	−0.1503	−0.1489	0.2442	0.2121
	(0.137)	(0.124)	(0.193)	(0.204)
SIZE	0.4828 ***	0.4712 ***	0.2590 ***	0.2343 **
	(0.158)	(0.139)	(0.094)	(0.108)
AGE	0.2595	0.2920	0.0181	0.3773
	(0.381)	(0.397)	(0.770)	(0.616)
Ownership	0.2109	0.2305	0.3824	0.2591 **
	(0.176)	(0.167)	(0.244)	(0.128)
BIGR	0.0060	0.0012	0.0111	0.0053
	(0.007)	(0.006)	(0.008)	(0.009)
Separation	−0.0060	−0.0026	−0.0039	−0.0104
	(0.007)	(0.006)	(0.010)	(0.009)
_cons	7.3563 *	7.4527 *	11.8092 **	9.8618 ***
	(4.195)	(4.015)	(4.987)	(3.759)
Firm FE	Yes	Yes	Yes	Yes
Year FE	Yes	Yes	Yes	Yes
N	536	604	662	594
R2	0.507	0.553	0.452	0.446
adj. R2	0.493	0.541	0.439	0.432

Notes: Column (1) presents the result of the TIS in the DSOM group (the selling expenses rate is higher than or equal to the industry average), Column (2) presents the result of the TIS in DSOM (the selling expenses rate is higher than or equal to the industry median), Column (3) is the results of the TIS in AM group (the selling expenses rate is lower than the industry average), and Column (4) is the results of the TIS in AM group (the selling expenses rate is lower than the industry median). Standard errors are clustered by firm level. ***, **, and * denote significance at 1%, 5%, and 10% level, respectively.

**Table 6:** Placebo Test.

	(1)	(2)
Variables	lnSE	lnSE
DID	−0.0300	0.0219
	(0.070)	(0.120)
ROA	1.8053	1.8140
	(1.294)	(1.311)
Tobin’s Q	−0.0213	−0.0226
	(0.016)	(0.020)
Lev	−0.0126	−0.0171
	(0.366)	(0.376)
Slack	−0.0103	−0.1123
	(0.271)	(0.274)
SIZE	0.3783 ***	0.3846 ***
	(0.072)	(0.070)
AGE	0.1350 ***	−0.5416 ***
	(0.023)	(0.113)
Ownership	0.4938	0.4631
	(0.322)	(0.323)
BIGR	0.0084	0.0076
	(0.007)	(0.007)
Separation	0.0160	0.0169 *
	(0.010)	(0.010)
_cons	9.0497 ***	12.6964 ***
	(1.420)	(1.543)
Firm FE	Yes	Yes
Year FE	No	Yes
N	1085	1085
R2	0.495	0.511
adj. R2	0.491	0.503

Notes: This table presents the results of placebo tests in the same regression sample in Columns (1) to (2), respectively. From Column (1) to Column (2), we add year fixed effect, in turn. Standard errors are clustered by firm level. ***, **, and * denote significance at 1%, 5%, and 10% level, respectively.

**Table 7:** Robustness Checks on Multi-period DID.

	(1)	(2)	(3)
Variables	lnSE	lnSE	lnSE
DID	0.3569 **	0.4140 *	0.4140 *
	(0.151)	(0.220)	(0.220)
ROA	1.0297 **	0.9488 **	0.9488 **
	(0.404)	(0.429)	(0.429)
Tobin’s Q	−0.0000	−0.0112	−0.0112
	(0.013)	(0.016)	(0.016)
Lev	0.6326 **	0.8115 ***	0.8115 ***
	(0.267)	(0.287)	(0.287)
Slack	−0.0556	0.0056	0.0056
	(0.156)	(0.161)	(0.161)
SIZE	0.4813 ***	0.5520 ***	0.5520 ***
	(0.086)	(0.092)	(0.092)
AGE	−0.6309 *	−0.8066 **	−0.8066 **
	(0.358)	(0.358)	(0.358)
Ownership	0.2955 *	0.1502	0.1502
	(0.165)	(0.169)	(0.169)
BIGR	0.0075	0.0066	0.0066
	(0.007)	(0.007)	(0.007)
Separation	0.0022	0.0016	0.0016
	(0.005)	(0.005)	(0.005)
_cons	10.7879 ***	17.4890 ***	10.5836 ***
	(2.543)	(5.155)	(2.539)
Firm FE	Yes	Yes	Yes
Year FE	Yes	No	Yes
Region FE	No	Yes	Yes
N	1614	1614	1614
R2	0.564	0.656	0.656
adj. R2	0.559	0.594	0.594

Notes: This table presents the results of multi-period DID in the same regression sample in Columns (1) to (3), respectively. From Column (1) to Column (3), we add year fixed effect, region fixed effect in turn. Standard errors are clustered by firm level. ***, **, and * denote significance at 1%, 5%, and 10% level, respectively.

**Table 8:** Robust Test: Alternative dependent variable.

	(1)	(2)
Variables	SEI	SEI
DID	0.0790 ***	0.0768 ***
	(0.012)	(0.016)
ROA	−0.2618 ***	−0.2370 ***
	(0.065)	(0.063)
Tobin’s Q	−0.0061 ***	−0.0038 **
	(0.002)	(0.002)
Lev	0.0264	0.0513
	(0.045)	(0.042)
Slack	0.0144	0.0185
	(0.030)	(0.029)
SIZE	−0.0306 **	−0.0345 **
	(0.014)	(0.015)
AGE	−0.0007	−0.2108 *
	(0.003)	(0.120)
Ownership	0.0244	0.0300
	(0.026)	(0.027)
BIGR	−0.0001	−0.0002
	(0.001)	(0.001)
Separation	0.0002	−0.0002
	(0.001)	(0.001)
_cons	0.9008 ***	2.4372 ***
	(0.300)	(0.905)
Firm FE	Yes	Yes
Year FE	No	Yes
N	1198	1198
R2	0.243	0.277
adj. R2	0.237	0.268

Notes: This table presents the results of the interaction term (DID) on the selling expenses intensity in the same regression sample in Columns (1) to (2), respectively. From Column (1) to Column (3), we add year fixed effect in turn. Standard errors are clustered by firm level. ***, **, and * denote significance at 1%, 5%, and 10% level, respectively.

**Table 9:** Robust Test: PSM-DID.

	(1)	(2)	(3)	(4)	(5)	(6)
Variables	lnSE	lnSE	lnSE	lnSE	lnSE	lnSE
DID	0.2987 *	0.3236 *	0.2773 *	0.2969 *	0.4587 ***	0.3756 **
	(0.151)	(0.180)	(0.150)	(0.178)	(0.117)	(0.166)
ROA	0.5937	0.7590	0.7388	0.9107	0.9006 **	1.0122 **
	(0.735)	(0.732)	(0.757)	(0.760)	(0.419)	(0.403)
Tobin’s Q	0.0197	0.0164	0.0199	0.0169	−0.0097	−0.0016
	(0.018)	(0.026)	(0.018)	(0.026)	(0.013)	(0.016)
Lev	0.2670	0.2986	0.3624	0.4108	1.0732***	1.1878***
	(0.403)	(0.419)	(0.412)	(0.429)	(0.317)	(0.328)
Slack	0.0901	0.1017	0.0859	0.1005	0.1342	0.1696
	(0.377)	(0.372)	(0.379)	(0.373)	(0.202)	(0.203)
SIZE	0.4451 ***	0.4687 ***	0.4597 ***	0.4823 ***	0.5011 ***	0.4509 ***
	(0.152)	(0.145)	(0.152)	(0.145)	(0.107)	(0.110)
AGE	0.0864 **	1.7821 ***	0.0844 **	1.6607 ***	0.0146	0.1567 *
	(0.036)	(0.563)	(0.035)	(0.547)	(0.026)	(0.087)
Ownership	1.0115	0.9740	0.9496	0.9081	0.7873 **	0.8595 **
	(0.810)	(0.834)	(0.810)	(0.835)	(0.325)	(0.335)
BIGR	0.0218	0.0202	0.0244	0.0227	0.0044	0.0027
	(0.017)	(0.017)	(0.017)	(0.017)	(0.011)	(0.011)
Separation	−0.0342	−0.0311	−0.0341	−0.0305	−0.0175	−0.0146
	(0.026)	(0.027)	(0.026)	(0.027)	(0.011)	(0.012)
_cons	7.8897 **	−0.3520	7.4862 **	−0.1837	7.5303 ***	7.8239 ***
	(3.190)	(3.773)	(3.195)	(3.733)	(2.200)	(2.301)
Firm FE	Yes	Yes	Yes	Yes	Yes	Yes
Year FE	No	Yes	No	Yes	No	Yes
N	358	358	359	359	729	729
R2	0.524	0.529	0.518	0.522	0.519	0.536
adj. R2	0.511	0.509	0.504	0.501	0.513	0.526

Notes: This table presents the results of the PSM-DID approach. From Column (1) to Column (2), we add year fixed effect in turn with one to four nearest-neighbor matching. From Column (3) to Column (4), we add year fixed effect in turn with caliper matching. From Column (5) to Column (6), we add year fixed effect in turn with kernel-matching method. Standard errors are clustered by firm level. ***, **, and * denote significance at 1%, 5%, and 10% level, respectively.

**Table 10:** Robust Test: Confounding Effects.

	(1)	(2)
Variables	lnSE	lnSE
DID	0.3689 ***	0.4481 **
	(0.080)	(0.173)
ROA	0.2545	0.3361
	(0.441)	(0.425)
Tobin’s Q	−0.0264 *	−0.0141
	(0.015)	(0.015)
Lev	0.8702 ***	1.0052 ***
	(0.292)	(0.288)
Slack	0.0166	0.0452
	(0.204)	(0.203)
SIZE	0.2563 **	0.2576 **
	(0.102)	(0.103)
AGE	0.0943 ***	−1.0584 ***
	(0.025)	(0.146)
Ownership	0.3499	0.3569
	(0.261)	(0.260)
BIGR	0.0051	0.0049
	(0.008)	(0.008)
Separation	0.0082	0.0052
	(0.007)	(0.008)
_cons	12.1702 ***	20.9179 ***
	(2.044)	(2.352)
Firm FE	Yes	Yes
Year FE	No	Yes
N	935	935
R2	0.511	0.524
adj. R2	0.506	0.517

Notes: This table presents the results of our baseline specification by excluding the samples belonging to the year 2020. From Column (1) to Column (2), we add year fixed effect in turn. Standard errors are clustered by firm level. ***, **, and * denote significance at 1%, 5%, and 10% level, respectively.

## Data Availability

The data that support the findings of this study are available from the CSMAR and Wind databases. Restrictions apply to the availability of these data, which were used under license for this study. Data are available with the permission of CSMAR and Wind.

## Appendix A

**Table A1:** Variable Description and Measurement.

Variable Type	Variables	Variable Name	Description
Dependent Variable	Selling expenses	SE	Log (1+ selling expenses)
Independent Variables	Treatment firm	Treat	Dummy variable, coded as 1 if treated, and 0 otherwise
	Treatment year	Post	Dummy variable, coded as 1 if a firm is observed after 2017
	DID	DID	Treat × Post
Control Variables	ROA	ROA	Net profit/Total asset
	Tobin’s Q	Tobin’s Q	Market value/Asset value
	Debt to asset ratio	Lev	Debt/Asset
	Slack resource	Slack	Cash and cash equivalents/ Asset value
	Firm size	SIZE	Natural logarithm of total asset
	Firm age	AGE	Time since listing of the company
	Firm ownership	Ownership	1, if the firm is a state-owned, 0 otherwise
	BIGR ratio	BIGR	Shareholding ratio of the largest shareholder
	Separation ratio	Separation	Difference between control and ownership of listed companies owned by actual controllers
